# Supplementary material for: Real-Time FTIR-ATR Spectroscopy for Monitoring Ethanolysis: Spectral Evaluation, Regression Modelling, and Molecular Insight
Source: Int J Mol Sci. 2025 Sep 25;26(19):9381. doi: 10.3390/ijms26199381 (PMC12524959; doi:10.3390/ijms26199381)
Supplement: Supplementary file 1 [file ijms-26-09381-s001.zip › ijms-3865198-supplementary.pdf]

Supplementary Material for

Real-time FTIR-ATR spectroscopy for monitoring ethanolysis: Spectral evaluation, regression modeling, and molecular in-sight

Jakub Husár, Lubomír Šánek, Jiří Pecha*

*Faculty of Applied Informatics, Tomas Bata University in Zlin,
Nad Stranemi 4511, 760 05 Zlin, Czech Republic*

**Corresponding author (husar@utb.cz)*

Abstract

As the demand for biodiesel continues to rise, there is a pressing need for efficient and continuous monitoring of the transesterification reaction at the industrial level. However, there is a lack of straightforward online monitoring methods capable of accurately fol-lowing the course of ethanolysis in various reaction conditions. In this work, simple linear regression (SLR) and multiple linear regression (MLR) models were developed to assess Fourier transform infrared spectroscopy (FTIR) data from a continuous flow cell, enabling real-time ethanolysis monitoring without sample pretreatment. Gas chromatography (GC) was utilised as the reference method to accurately characterise the reaction mixture's composition during ethanolysis. Extensive correlation analysis was performed to identify spectra regions where the reaction system's state changes are observable. The gained re-gions were subsequently applied in the linear regression model's development. This novel approach resulted in the performance of simple linear regression comparable to complex partial least squares (PLS) regression model (RMSEP = 2.11). The developed online moni-toring system was validated in a wide range of reaction conditions (40-60°C; 0.25-1.0% w/w NaOH); it effectively identifies dynamic changes in the ethanolysis process and con-firms achieving the threshold value of ester content set by EU regulation directly in the production process.

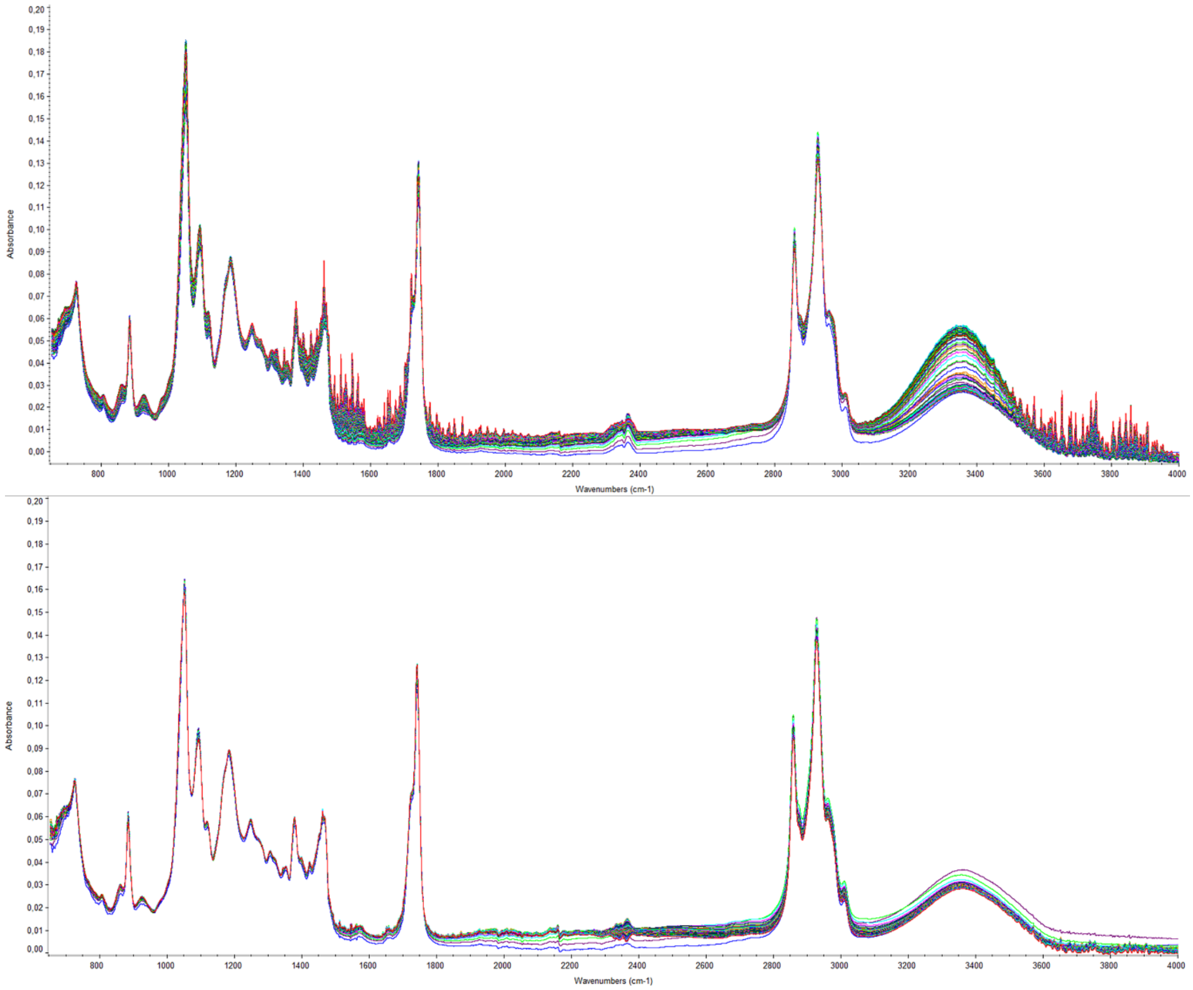

Figure S1. Two experiments with visible different spectra at the beginning (blue) and visible effect of the water vapour in the spectra (1200-2200, 3400+ cm^{-1})

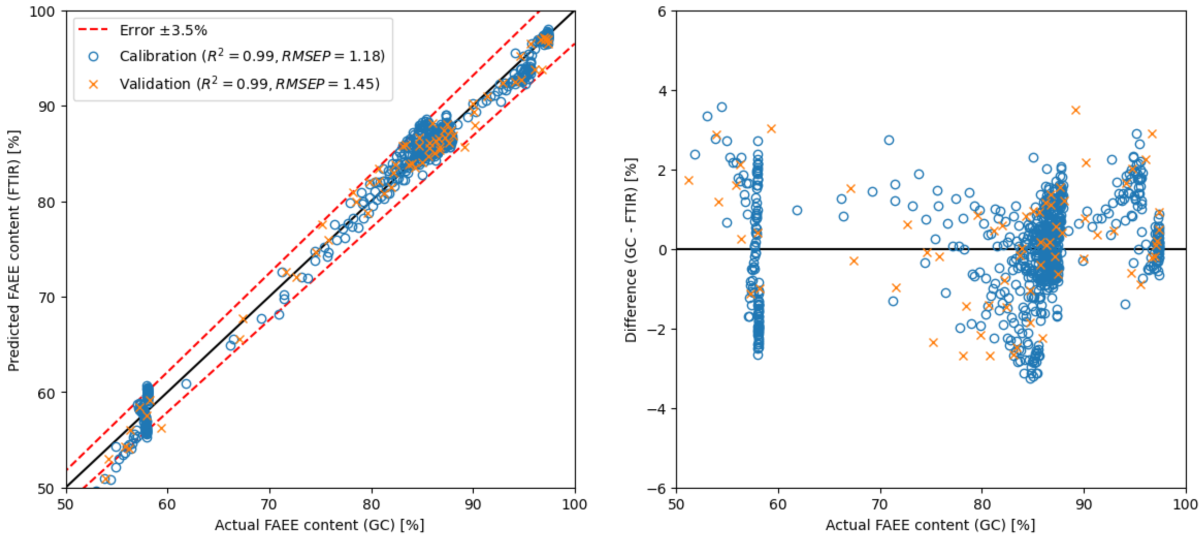

Figure S2. Calibration and validation of MLR model using 7 regions from spectra

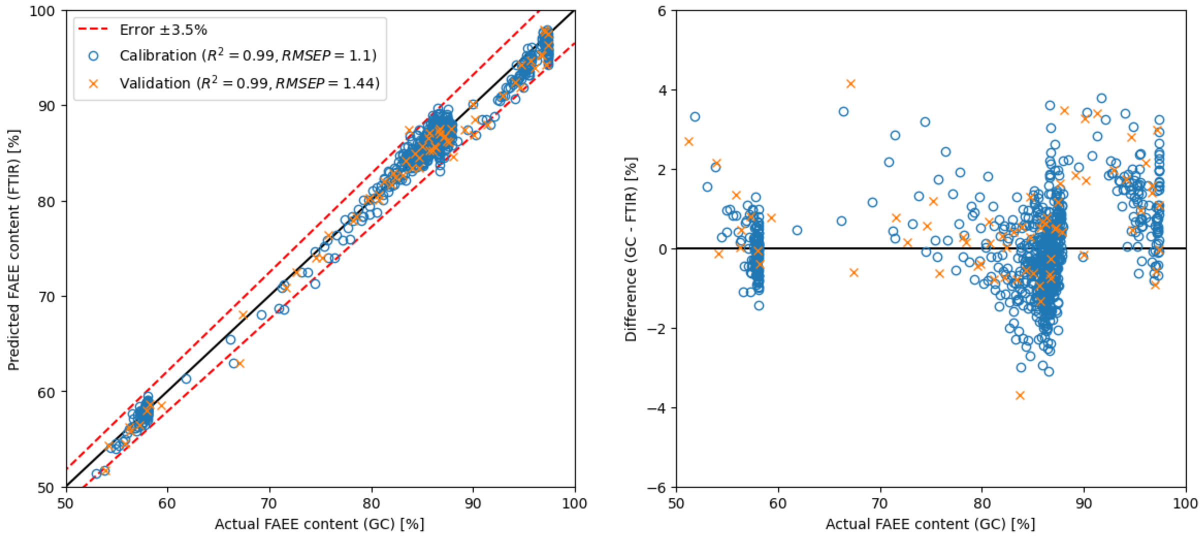

Figure S3. Calibration and validation of MLR model using 9 regions from spectra derivative
